# Sequential treatment with ipilimumab and BRAF inhibitors in patients with metastatic melanoma: data from the Italian ipilimumab expanded access programme (EAP)

**DOI:** 10.1186/2051-1426-1-S1-P69

**Published:** 2013-11-07

**Authors:** Paolo Antonio Ascierto, E Simeone, V Chiarion Sileni, P Queirolo, M Del Vecchio, L Di Guardo, M Guidoboni, P Marchetti, GC Antonini Cappellini, PF Ferrucci, F Cognetti, MG Bernengo, M Guida, R Marconcini, M Mandalà, C Cimminiello, G Rinaldi, M Aglietta, L Calabrò, M Maio

**Affiliations:** 1 Istituto Nazionale Tumori Fondazione "G. Pascale", Naples, Italy; 2 Institute of Oncology IOV-IRCCS, Padua, Italy; 3 San Martino Hospital-National Institute for Cancer Research, Genoa, Italy; 4 National Cancer Institute, Milan, Italy; 5 Romagna National Cancer Institute, Meldola, Italy; 6 Dermathopatic Institute of the Immaculate, Rome, Italy; 7 Sant'Andrea Hospital, University Sapienza, Rome, Italy; 8 Istituto Europeo di Oncologia, Milan, Italy; 9 Regina Elena National Cancer Institute, Rome, Italy; 10 University Hospital St John the Baptist, Turin, Italy; 11 National Cancer Research Center "Giovanni Paolo II", Bari, Italy; 12 "Gathered Hospitals of Santa Chiara", Pisa, Italy; 13 Papa Giovanni XXIII Hospital, Bergamo, Italy; 14 San Raffaele Hospital, Milan, Italy; 15 Paolo Giaccone Polyclinc University Hospital, Palermo, Italy; 16 Piedmont Oncology Foundation, Candiolo, Italy; 17 Hosptal of Siena Istituto Toscano Tumori, Siena, Italy

## Background

Data to guide the order in which ipilimumab and vemurafenib are used in patients with advanced melanoma are limited. Here are reported outcomes from patients treated in the ipilimumab EAP who received both drugs.

## Methods

Patients with pretreated, BRAFV600 mutation-positive advanced melanoma who had received BRAF inhibitor before or after ipilimumab were eligible for analysis.

## Results

93 patients were eligible: 48 patients received a BRAF inhibitor after ipilimumab and 45 patients ipilimumab after a BRAF inhibitor. Median overall survival (OS) was 14.5 and 9.9 months for the two groups, respectively (P=0.04). Among patients who received a BRAF inhibitor first, 18 (40%) had rapid disease progression and were unable to complete ipilimumab treatment as for protocol (rapid progressors). For this group median OS from the cessation of treatment with a BRAF inhibitor was 1.2 months. 27 patients had slower disease progression and were able to complete all four doses of ipilimumab (slow progressors); median OS was significantly longer (12.7 months; P<0.0001).Younger age and the presence of brain metastasis were significantly associated with a poorer outcome (P=0.02).

## Conclusions

This EAP data suggests that pretreated, BRAF-mutated patients who have rapid disease progression upon failing treatment with a BRAF inhibitor die in one month, so they may benefit from receiving ipilimumab as the first part of their sequential regimen, otherwise clinical benefit may be limited due to them not being able to receive the full induction treatment.

